# Supplementary material for: Long- and Short-Term Conductance Control of Artificial Polymer Wire Synapses
Source: Polymers (Basel). 2021 Jan 19;13(2):312. doi: 10.3390/polym13020312 (PMC7835966; doi:10.3390/polym13020312)
Supplement: Supplementary file 1 [file polymers-13-00312-s001.pdf]

Supplementary Materials for

Long-term and Short-term Conductance Control of the Artificial Polymer Wire Synapse

Naruki Hagiwara [1], Shoma Sekizaki [1], Yuji Kuwahara [1], Tetsuya Asai [2] and Megumi Akai-Kasaya [1,2,*]

[1] *Graduate School of Engineering, Osaka University, Osaka 565-0871, Japan;*

[2] *Faculty of Information Science and Technology, Hokkaido University, Sapporo 060-0814, Japan;*

* E-mail: akai@ist.hokudai.ac.jp

Contents:

Figure S1. **Condition of applying voltage for dendritic PEDOT:PSS electrodeposition**

Figure S2. **Voltage pulse interval dependence of conductive polymer wire diameter distribution change**

Figure S3. **The transition from LTP to STP**

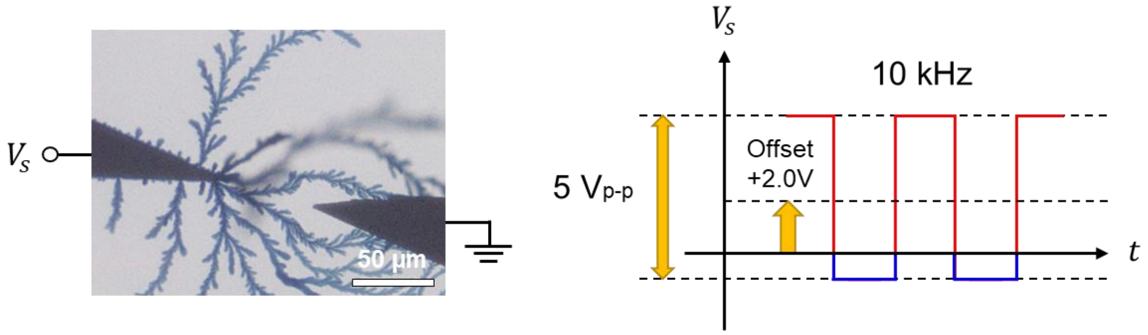

Figure S1. Condition of applying voltage for dendritic PEDOT:PSS electrodeposition. Bipolar square-wave AC voltage (10 kHz, 5 $V_{p\text{-}p}$) with an offset of 2 V was used. By adding an offset, the dendritic PEDOT: PSS grew quickly from one electrode.

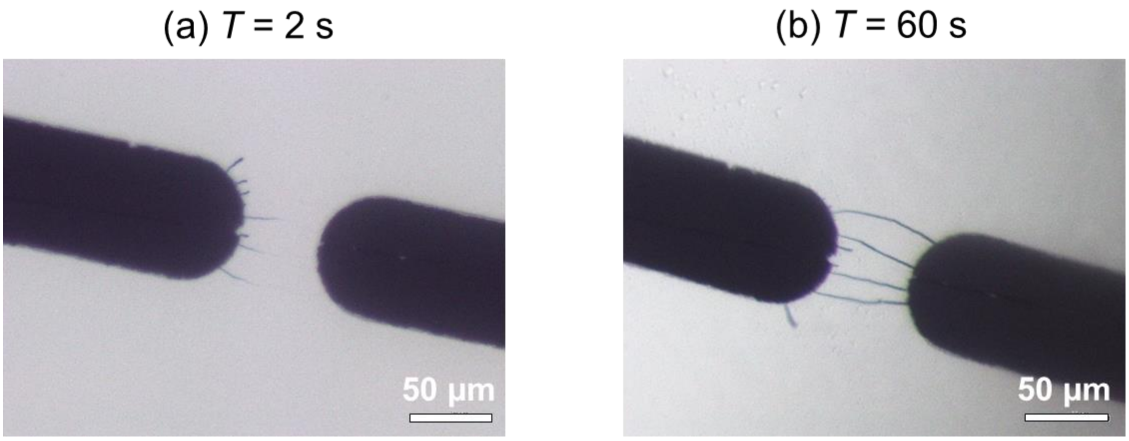

Figure S2. Voltage pulse interval dependence of conductive polymer wire diameter distribution change. Optical microscopic images show the electrode gap crosslinked by conductive polymer wires after voltage pulsing (V = 2.5V, W = 10 ms) at (a) T = 2 s and (b) T = 60 s, respectively. When pulses with short intervals were applied, only the anode side of wire became thicker, resulting in an asymmetric wire. On the other hand, pulses with long intervals led to a uniformly thicker wires

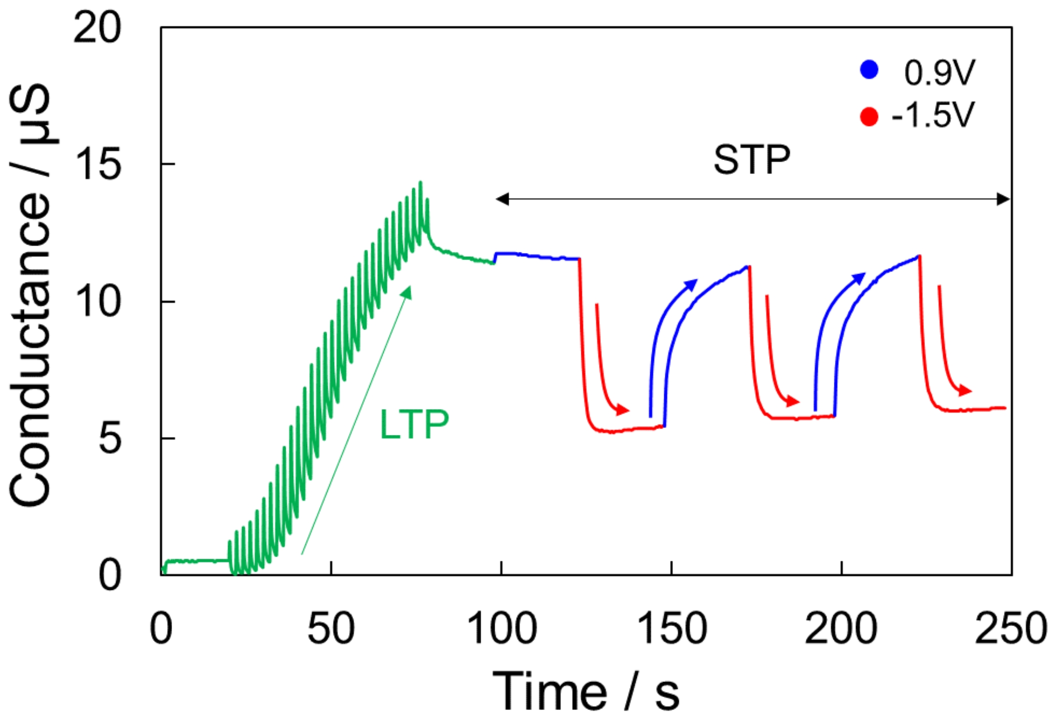

Figure S3. The transition from LTP to STP. After LTP was induced by voltage pulsing (V = 2.5V, W = 10 ms, T = 2 s, 30 times), subsequent STP was induced by voltage pulseing (V = 0.9 or -1.5 V, W = 10 ms, T = 0.5 s)
